# Pilot-Scale Acidogenic Fermentation of Reground Pasta Byproduct for Polyhydroxyalkanoate Production with Mixed Microbial Cultures

**DOI:** 10.1021/acssuschemeng.4c03754

**Published:** 2025-02-20

**Authors:** Gaia Salvatori, Angela Marchetti, Anna Maria Russo, Jesus Rodriguez, Vadim Scerbacov, Francesco Fianelli, Sara Alfano, Simona Crognale, Alessio Massimi, Simona Rossetti, Giacomo Canali, Tiziana De Micheli, David Bolzonella, Marianna Villano

**Affiliations:** a Department of Chemistry, Sapienza University of Rome, P.le Aldo Moro 5, Rome 00185, Italy; b InnovEn Srl, Verona, 37134, Italy; c Water Research Institute (IRSA), National Research Council (CNR), Via Salaria km 29300, Monterotondo, Rome 00015, Italy; d National Biodiversity Future Center (NBFC), Palermo 90133, Italy; e Barilla G. e R. Fratelli − Società per Azioni − Socio Unico, Via Mantova 166, Parma 43122, Italy; f Department of Biotechnology, University of Verona, Via Strada Le Grazie 15, Verona 37134, Italy; g Research Center for Applied Sciences to the Safeguard of Environment and Cultural Heritage (CIABC), Sapienza University of Rome, P.le Aldo Moro 5, Rome 00185, Italy

**Keywords:** acidogenic fermentation, reground pasta, pilot scale, mixed microbial cultures, polyhydroxyalkanoates, terpolymer

## Abstract

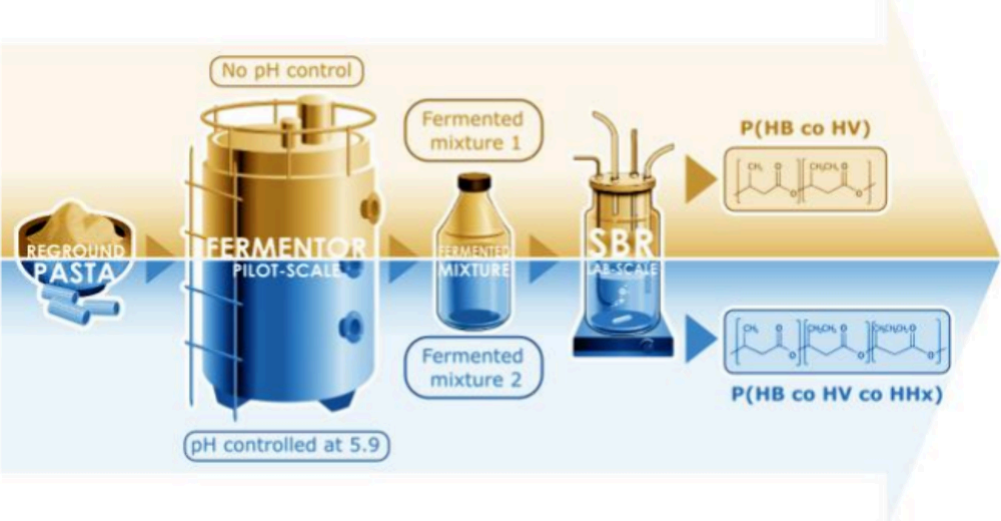

The production of polyhydroxyalkanoates (PHAs) has been herein investigated by using an organic acid mixture originated from a pilot-scale acidogenic fermentation (AF) of reground pasta (RP) byproduct. The pilot-scale AF process was conducted either under no pH control or with the pH maintained at a value of 5.90, with the two obtained fermented mixtures termed RP-fermented 1 and RP-fermented 2, respectively. The fermented mixtures were fed to a lab-scale sequencing batch reactor (SBR), operated at short hydraulic retention time (HRT, 0.5 days) and sludge retention time (SRT, 1 day) and at two values of the applied organic loading rate (OLR) of 2.12 gCOD_ACIDS_/Ld and 4.25 gCOD_ACIDS_/Ld. During all of the SBR operating conditions, a high selective microbial pressure was established, as confirmed by both the microbiology analysis and the detected values of the storage yield (which reached a maximum value of 0.68 ± 0.04 COD_PHA_/COD_ACIDS_). A poly(hydroxybutyrate/hydroxyvalerate) copolymer and a poly(hydroxybutyrate/hydroxyvalerate/hydroxyhexanoate) terpolymer were produced with the RP-fermented 1 and RP-fermented 2 streams, respectively. When the OLR of 2.12 gCOD_ACIDS_/Ld was applied to the SBR, the stored copolymer and terpolymer presented very similar molecular weights of 339 and 389 kDa, respectively.

## Abstract 2

Reground pasta byproducts serve as renewable feedstock for producing polyhydroxyalkanoates with varying monomer compositions using mixed microbial cultures

## 1 Introduction

Nowadays, fossil-based plastic materials are still largely widespread despite the awareness of their direct climate impact on global greenhouse gas (GHG) emissions related to their production processes. Presently, plastic production is estimated at 460 Mt and their end-of-life management is extremely challenging.^1^ In the last decades, besides the implementation of regulations on plastic recycling, much attention has also turned to the search for alternative sustainable materials, such as bioplastics with physical–chemical properties similar to those of conventional plastics but with the advantage of being biobased and, in some cases, also truly biodegradable.^2^ In this perspective, PHAs represent optimal candidates to replace petroleum-based polymers, since they are a family of linear polyesters stored by over 300 species of microorganisms,^3^ can be produced from renewable sources,^4,5^ and are fully biodegradable in the environment.^6^ To overcome the high production cost, mainly due to the use of pure microbial cultures and to the maintenance of sterile operating conditions,^7^ the employment of mixed microbial cultures (MMCs)—combined with the use of agro-industrial organic wastes and byproducts as feedstock—is being investigated.^8,9^ Along this line, the recent European policies on circular economy encourage the use of secondary raw materials or byproducts to enhance the transition from a linear toward a circular economy.

A wide range of wastes and byproducts have been tested and considered suitable feedstocks for MMC-PHA production and accumulation, including cheese whey, olive oil mill wastewater, grapes, fruit or juice from fruit pulp, urban wastes, sewage sludge, and farinaceous byproducts.^4,9−11^ More in detail, when dealing with MMC, the PHA production process involves the combination of the feedstock AF step and the PHA production stages, which comprise the microbial selection into PHA-storing microorganisms as well as the polymer accumulation.^12,13^ The first step aims to obtain a mixture enriched with carboxylic acids, which are the main precursors for MMC-PHA synthesis in the following process stages. In particular, the composition of the obtained fermented mixture strongly affects the final PHA composition. As an example, when acetate is used as a unique carbon source, poly(3-hydroxybutyrate) (P3HB) is produced, which is the most common polymer of the PHA family and its properties are comparable to those of polypropylene (fossil-based) but with a strong limitation in terms of the lower extension to break and higher crystallinity.^14,15^ A mixture of butyrate, propionate, and valerate can be converted directly to 3-hydroxybutyryl-CoA and 3-hydroxyvaleryl-CoA,^16^ which form the 3HB and the 3-hydroxyvalerate (3HV) monomer, respectively. The 3HV presence in the poly(3-hydroxybytyrate-3-hydroxyvalerate), P(3HB-*co*-3HV), copolymer affects its elastomeric properties, depending on the HV content.^17^ The copolymer production has been widely tested using waste feedstocks. As an example, the codigestion of the organic fraction of municipal solid waste (OFMSW) with sewage sludge has been found to produce the P(3HB-*co*-3HV) copolymer achieving high performance in terms of storage capacity (0.78 gPHBV/g volatile suspended solids).^18^ Recently, with fruit waste, a terpolymer containing hydroxyhexanoate (HHx) at a composition of 33/1/66 (HB/HV/HHx, % wt/wt) has been obtained achieving a maximum PHA content of 71.3% (wt/wt).^9^ In general, the use of organic waste streams allows reducing the overall PHA production cost, of which the feedstock typically represents up to around 50% of the total.^19^

This study aims to assess the feasibility of MMC-PHA production by using RP, which is a byproduct deriving from the production of dried pasta, the second most consumed food worldwide prevalently constituted by carbohydrates and proteins.^20,21^ RP is currently mainly used for animal feed,^22^ and it has been recently tested for PHA production with phototrophic purple bacteria,^23^ but limited studies are still available for its possible conversion into biopolymers especially with reference to the use of MMC. A previous study pointed out the high potential of RP byproduct to be converted into carboxylic acids (up to approximately 55%), which are the most suitable substrates for MMC-PHA production, through batch AF tests.^24^ The process herein proposed integrates the RP AF, operated for the first time on a pilot scale, with the microbial selection stage operated on a laboratory scale.

Two different operating conditions (no pH control and pH control at 5.90) were tested to evaluate the optimal acidification degree. The two obtained fermented streams were used as feedstocks to a lab-scale SBR, wherein characteristic parameters (i.e., intracellular polymer content and storage yield) as well as polymer composition were adopted to assess the process performance.

## 2 Materials and Methods

### 2.1 Pilot-Scale AF of RP Byproduct

RP, provided by the same food industry involved in its generation, was the agro-industrial byproduct herein used as feedstock during the AF process for the production of carboxylic acids to be converted into PHA with MMC.

AF was performed in a pilot-scale plant made of several units and automatically controlled by a programmable logic controlled (PLC) board (Easy access 2.0). The pilot plant was hosted at the “La Torre” farm, located in “Isola della Scala” (Verona, Italy), and its main units comprised a feedstock storage tank (maximum volume of 5 m^3^), equipped with a stirrer and a crushing recirculation pump also used for the fermentor feeding, and a fermentation unit (also referred to as bioreactor). The bioreactor was made of AISI 316 stainless steel with a maximum operating capacity of 5 m^3^. The bioreactor was equipped with a stirrer, and the internal temperature was kept constant at 37 °C through the external double jacket of the fermentor. The anaerobic fermented stream underwent a solid/liquid separation step, and the liquid fraction was then treated in a dynamic tangential ultrafiltration unit (JU.CLA.S, Juice Clarification Systems, model: MMSL LAB, Italy), equipped with a 0.2 μm ceramic disk sized to produce an ultrafiltered fermentation liquid (UF-FL) with a permeate flow capacity of 15 L/h. The digestate deriving from the local full-scale anaerobic digestion plant treating livestock manure and agro-waste, located in Isola della Scala, was used as the inoculum of the anaerobic bioreactor.

The compositions in terms of total solid (TS), total volatile solid (TVS), and chemical oxygen demand (COD) of both RP and digestate are reported in Table 1.

**Table 1:** TS, TVS, and COD Content in the RP Byproduct and in the Digestate

	**RP**(kg/tons)	**digestate** (kg/m^3^)
TS	915.4 ± 4.8	121.2 ± 2.6
TVS	744.4 ± 11.2	105.1 ± 4.2
COD	968.8 ± 12.5	100.7 ± 8.4

The feedstock was prepared by mixing water, digestate, and RP in the mixing tank at a ratio of 3:4:1 (v/v). After mixing, the composition of the resulting solution accounted for 135.3 ± 2.6 kgTS/m^3^, 113.1 ± 4.2 kgTVS/m^3^, and 132.0 ± 6.5 kgCOD/m^3^.

The AF bioreactor was daily fed with 0.8 m^3^ of the mixed feedstock resulting in an OLR of 18.08 kgTVS/m^3^day and a hydraulic retention time (HRT) of 6.25 days. The bioreactor, operated for about 150 days, was kept under constant agitation to promote hydrolysis and the AF process. During the first operating period (from day 0 to day 60), the pH was not controlled, resulting in the establishment of rather acidic conditions (pH equal to 4.20 ± 0.25). In the second operating period (from day 61 to day 150), the fermentor was reinoculated with fresh digestate, and an additional buffer capacity was provided in order to maintain the pH value at 5.90. The two obtained fermented effluents were used as feedstocks for PHA production at the lab-scale.

### 2.2 Laboratory-Scale PHA Production in a SBR

The fermented mixtures obtained at the pilot scale were fed to the lab-scale SBR (1 L working volume), aimed at the microbial selection into PHA-storing microorganisms through the application of the “feast/famine” (F/F) regime.^25,26^ The SBR inoculum consisted of activated sludge collected from a full-scale municipal wastewater treatment plant, and the reactor was fed with the RP-fermented streams at two applied OLRs, 2.12 and 4.25 gCOD_ACIDS_/Ld.

Herein, the OLR refers to the concentration of carboxylic acids contained in the RP-fermented solutions, and it was changed by changing the COD concentration in the SBR feeding mixture by diluting the fermented streams with a mineral medium containing a phosphate buffer. The composition of the mineral medium is reported elsewhere^27^ except for the ammonia source, which was not provided since the fermented mixtures already contained ammonia at a concentration of about 2.6 g/L. During the first 25 days of the SBR operation, the HRT corresponded to the SRT and was equal to 1 day. Subsequently, a settling phase was included in the SBR cycle, resulting in an HRT equal to 0.5 days and an SRT equal to 1 day. The length of the SBR working cycle was fixed at 6 h consisting of an aerated feeding phase (12 min, 0.50 L/cycle corresponding to 2 L/day), aeration (50 min), settling (40 min), separate discharge of settled biomass (3 min, 0.10 L/cycle corresponding to 0.40 L/day) and supernatant (2 min, 0.40 L/cycle corresponding to 1.60 L/day) without air supply, and reaeration (253 min) (Figure S1). The feast and famine phases were conducted in a reactor volume of 1 and 0.50 L, respectively; for this reason, throughout the manuscript, all values were expressed in grams. This approach was adopted as a strategy to minimize the competitive growth of non-PHA-storing microorganisms throughout the SBR cycle, which can particularly occur when dealing with a complex feedstock. Indeed, the latter typically includes both readily biodegradable COD (rbCOD) and slowly biodegradable COD (sbCOD). Even though the rbCOD is depleted and stored as intracellular PHA during the feast phase, the residual fraction of the sbCOD can be used for microbial growth during the famine phase. The inclusion of a settling phase, after the end of the feast phase, has been demonstrated as an efficient tool to enhance the microbial selection through the withdrawal of most of the residual sbCOD contained in the clarified supernatant.^28^

The pH of the reactor was between 7 and 8 throughout the working cycle, and the temperature was maintained at 25 °C using a thermostat jacket. During each cycle, a continuous aeration was provided through an air supply by means of ceramic diffusers and the mixing was provided with a mechanical impeller. The SBR performance was characterized by determining the total and volatile suspended solids concentration (TSS and VSS, respectively), as well as the PHA, COD, carboxylic acids, and ammonia concentrations.

### 2.3 Analytical Methods

The initial mixture of RP byproduct contained in the storage tank of the pilot plant was monitored in terms of TS and TVS once per week throughout the experimentation period, according to the standard method APHA.^29^ Likewise, the RP-fermented stream was characterized two or three times per week to measure its pH value as well as the COD, carboxylic acids, and ammonia concentrations. Ammonia was quantified by the Nessler spectrophotometric method, and the absorbance of reacted samples was measured at 420 nm wavelength (SHIMADZU Spectrophotometer UV-1800).^29^ COD was measured by a colorimetric method, involving the use of a commercial kit (Tube test NANOCOLOR COD 25–1500 mg/L, Merck). The concentration of microorganisms, reported as VSS, was determined according to the standard method APHA.^29^ The concentration of carboxylic acids was determined through gas chromatographic analysis by injecting 1 μL volume of liquid phase in an Agilent GC 8860 instrument equipped with a DB-FFAP column (30 m × 530 μm × 0.25 μm), N_2_ was used as carrier gas at a flow rate of 30 mL/min, the initial oven temperature was set at 60 °C, and it was increased up to 175 °C at a rate of 20 °C/min; the flame ionization detector (FID) was set at 320 °C. The PHA concentration was quantified by a GC method injecting 1 μL volume of organic liquid phase into an Agilent 8860 GC equipped with a HP-5 column (30 m × 320 μm × 0.25 μm), N_2_ was used as carrier gas at a flow rate of 10 mL/min, the oven temperature was initially maintained at 100 °C for 1 min; then increased up to 130 °C at a rate of 12 °C/min and further increased up to 250 °C at a rate of 25 °C/min; the FID was maintained at 300 °C. Samples for PHA analysis were prepared as described elsewhere.^30^ The PHA-rich biomass collected from the SBR was extracted with chloroform as reported in Salvatori et al.^31^ and was characterized in terms of purity (PHA, % wt/wt), composition, and molecular weight. Since the investigation of alternative and more environmentally acceptable methods for PHA recovery from MMC was not the main aim of this study, chloroform has been used here as the extraction solvent according to the benchmark procedure. The average molecular weight (*M_w_*) and polydispersity index (PDI = *M_w_*/*M_n_*) of the extracted P(3HB-co-3HV) and P(3HB-co-3HV-co-3HHx) polymers were determined by using a gel permeation chromatography (GPC) equipped with a pump (JASCO PU-4180), a guard column and two columns in series (TSKgelG6000-HHR and TSKGel GMHHR-H), a column oven (JASCO CO-4060), and a refractive index detector (JASCO RI-4030), as reported elsewhere.^32^

### 2.4 Calculations

Several parameters were used to characterize the SBR performance. In particular, the nonpolymer biomass (or active biomass, *X_A_*) was calculated as the difference between the VSS and PHA quantities detected at different moments of the SBR cycle: *X_A_* (mg) = VSS (mg) – PHA (mg). This value was converted into COD, according to a conversion factor of 1.42 mg COD/mg *X_A_*.^33^ The intracellular PHA content was defined as the ratio between the PHA and VSS concentrations (%, wt_PHA_/wt_VSS_). For the yield calculation, the mass values of PHA and carboxylic acids were converted into COD units by using the relative conversion factor from oxidation stoichiometry.^33^ The storage yields were defined as the ratio between the produced polymer (ΔCOD_PHA_) and the consumed substrate, which was referred to as the total soluble organic matter (−Δ_S_COD_TOT_) or to the consumed acids (−ΔCOD_ACIDS_). The growth yield was calculated as the ratio between the active biomass (*X_A_*) produced and the removed substrate (as COD_ACIDS_ or _S_COD_TOT_), as given in the following equations:
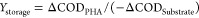
1
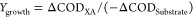
2

The specific overall substrate removal rate (−*q*_*S*_^end of feeding phase^) was calculated per unit of active biomass concentration in correspondence to the beginning of the feeding phase (*X_A_* expressed as COD), and the duration of the feeding phase (*t*):
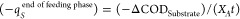
3

Also, specific substrate removal rates were calculated with reference to the specific consumption rates of individual acids (i.e., acetic, propionic, butyric, valeric, and caproic acid). The content of PHA monomers in the produced copolymer or terpolymer was defined as the ratio between HB, HV, or HHx and the overall polymer concentration, according to
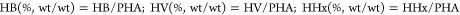


### 2.5 Microbiological Characterization

Inoculum and aerobic sludge samples (10 mL) were taken over the SBR operation. A small aliquot (2 mL) was centrifuged at 15,000 rpm for 2 min, and the resulting pellet was used for DNA extraction by DNeasy PowerSoil Pro Kit (QIAGEN, Italy) following the manufacturer’s instructions.

The extracted DNA was utilized as the template for the amplification and high-throughput sequencing on MiSeq platform (Illumina, USA) of the V1–V3 region of bacterial 16S rRNA gene (27F 5′-AGAGTTTGATCCTGGCTCAG-3′; 534R 5′-ATTACCGCGGCTGCTGG-3′) following the procedure described in Crognale et al.^34^

The raw sequences were quality assessed with Fastqc software and then analyzed using QIIME2 v. 2018.2 as described in Crognale et al.^34^ Taxonomy was assigned to amplicon sequence variants (ASVs) using a pretrained naïve-Bayes classifier based on the 16S rRNA gene database at 99% similarity to the Silva132 release.^35^ High-throughput sequencing of the V1–V3 region of the bacterial 16S rRNA gene yielded a total of 67^.^104 sequence reads after quality control and bioinformatic processing.

Based on the taxonomical classification, the relative percentage of bacterial genera described in the literature for their capability to accumulate PHA was calculated for each sample and was reported in the text as “putative PHA-storing bacteria abundance”. The data set is available through the Sequence Read Archive (SRA) under accession number PRJNA1036813.

The genomic DNA was also used as the template for the quantification of total bacterial load by the 16S rRNA gene and of a functional gene involved in the PHA synthesis (*phaC* gene). Absolute quantification assays were performed via digital droplet PCR (ddPCR) with the QX200 Droplet Digital PCR System (Bio-Rad, United States) as described elsewhere.^36^ The primer sets used were BAC1055F (5′-ATGGCTGTCGTCAGGT-3′)/BAC1392 (5′-ACGGGCGGTGTGTAC-3′) for 16S rRNA^37^ and CF1 (5′-ATCAACAARTWCTACRTCYTSGACCT-3′)/CR4 (5′- AGGTAGTTGTYGACSMMRTAGKTCCA-3′) for targeting the type I and II *phaC* gene-encoding PHA synthases.^26^

Data were analyzed using the QuantaSoft software (Bio-Rad, United States) by calculating the ratio of the positive droplets over the total droplets in each sample. Quantitative data have been reported as gene copies/gVSS (95% confidence intervals); the abundance of *phaC* gene was also normalized using 16S rRNA as a housekeeping gene.

## 3 Results and Discussion

### 3.1 Pilot-Scale AF under Different Operating Conditions

The main characteristics of the RP-fermented streams are listed in Table 2. In particular, the total soluble COD (_S_COD_TOT_) was monitored twice a week, accounting for average values of 71.7 ± 2.1 and 86.9 ± 6.0 (g/L) for RP-fermented 1 (collected during the first operating period with no pH control) and RP-fermented 2 (collected during the second operating period with the pH maintained at 5.90), respectively. The acidification degree was around 50 and 75% (wt/wt) for RP-fermented 1 and RP-fermented 2, respectively. This parameter represents the fraction of _S_COD_TOT_ consisting of acids (COD_ACIDS_), and it is useful for assessing the AF performance since its high value indicates a correct and balanced fermentation process. In general, fermentation compounds other than acids can be contained in fermented streams, such as alcohols, among which ethanol has been previously reported to be converted into biopolymers with MMC.^38^ However, here, the main attention has been paid to characterizing the RP-fermented solutions in terms of carboxylic acid composition which, in turn, affects the PHA composition. In this context, the possibility of managing the distribution of carboxylic acids by monitoring the pH of the fermentor is relevant for the following stages of microbial selection and polymer accumulation. During the first operating period, the pH was stabilized at an average value of 4.20, and the RP-fermented 1 was characterized by a lower acid content with respect to the _S_COD_TOT_ than the second operating period (RP-fermented 2) (Table 2). These results clearly indicate that controlling the pH at a value higher than 4.50 led to a greater RP conversion into carboxylic acids. In the second operating period, the fermentor was reinoculated with fresh digestate, which positively affected the fermentation yield. Indeed, as reported in the literature, pH values higher than 4.80 promoted a higher fatty acid concentration in the fermentation broth.^10^ Furthermore, additional buffer capacity was provided by adding sodium bicarbonate (NaHCO_3_) to the fermentor (about 5.6 kg/day, once a day) in order to stabilize the pH at a value of 5.90 and to make a preliminary evaluation of the effect of the pH on the RP fermentation performance. However, further process optimization is still warranted in order to minimize operational costs associated with the addition of chemical compounds, including sodium bicarbonate (whose commercial cost accounts for around 0.52 $/kg).

**Table 2:** Characterization of the RP-Fermented Mixtures Obtained during the Operation of the Pilot-Scale Fermentor

**fermented mixtures**	_**S**_**COD**_**TOT**_ (gCOD/L)	**COD**_**ACIDS**_**/**_**S**_**COD**_**TOT**_ (%, wt/wt)	**Even C (C**_**2**_**–C**_**4**_****–**C**_**6**_**)**(%, wt/wt)	**Odd C (C**_**3**_**–C**_**5**_**)**(%, wt/wt)	**N-NH**_**4**_^**+**^(g/L)	**pH**
RP-fermented 1 (days: 0–60)	71.7 ± 2.1	49.8 ± 1.7	64.3 ± 0.7	35.7 ± 0.7	2.5 ± 0.1	4.2 ± 0.3
RP-fermented 2 (days: 61–150)	86.9 ± 6.0	75.1 ± 1.2	74.4 ± 0.3	25.9 ± 0.4	2.6 ± 0.2	5.9 ± 0.2

**Figure 1:**
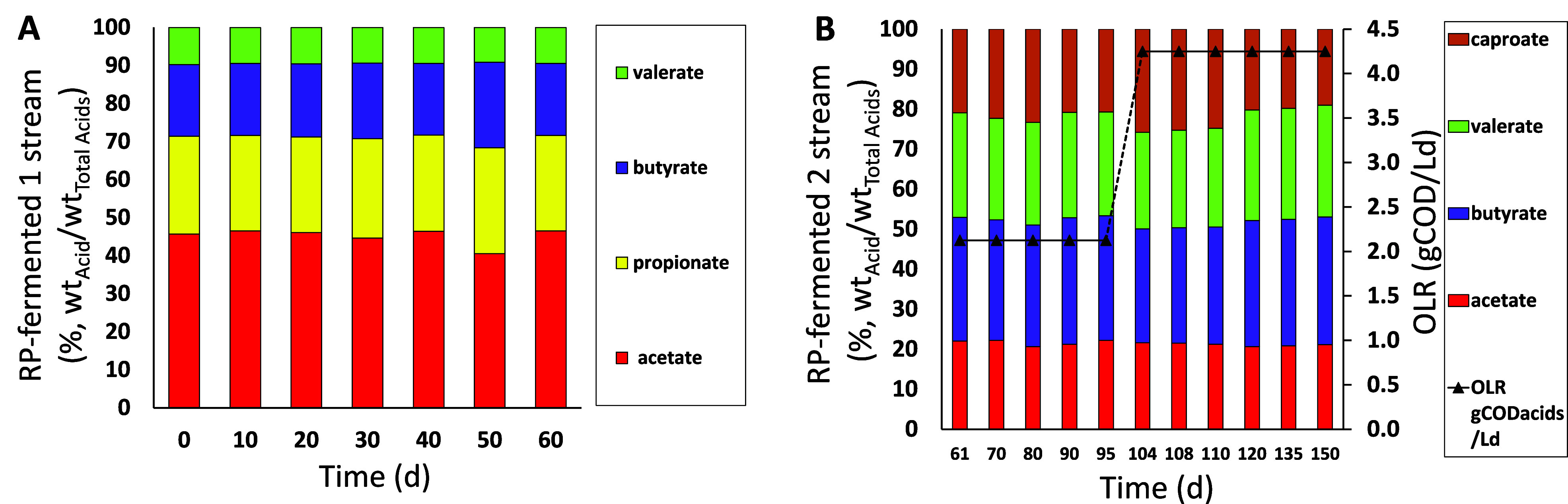
Composition of carboxylic acids for the RP-fermented 1 (A) and RP-fermented 2 (B) mixtures. Time on the *x*-axis refers to the operational days of the SBR fed with one of the two fermented streams.

Figure 1 shows the trend of the organic acid composition in the RP-fermented streams used as feedstock of the lab-scale SBR. In particular, Figure 1A refers to the stream collected during the first operating period of the pilot-scale bioreactor and fed to the SBR during its first 60 operational days. This stream was characterized by the presence of short-chain carboxylic acids (SCAs) with both even (C2 and C4) and odd (C3 and C5) number of carbon atoms, which are precursors for the synthesis of both the 3HB and 3HV monomers.^16^Figure 1B refers to the single-acid composition of the RP-fermented 2 stream wherein the absence of propionic acid and the presence of caproic acid (on average 22 ± 1%, wt/wt of the total acids) were likely due to the pH increase in the pilot-scale bioreactor. This stream was fed to the SBR from day 61 of operation until the end of the experimentation.

### 3.2 Microbial Selection in the Lab-Scale SBR Fed with RP-Fermented 1 at OLR of 2.12 gCOD_ACIDS_/Ld

The microbial selection toward PHA-storing microorganisms was performed in an SBR inoculated with activated sludge and functioned at different operating conditions, as summarized in Table 3.

**Table 3:** Operating Conditions of the SBR fed with the RP-Fermented Streams

**feedstock**	**operation days**	**OLR(gCOD**_**ACIDS**_**/L d)**	**HRT (d)**	**SRT (d)**
RP-fermented 1	run1 (from day 0 to day 60)	2.12	0.5	1
RP-fermented 2	run1 (from day 61 to day 103)	2.12	0.5	1
	run2 (from day 104 to day 160)	4.25		

**Figure 2:**
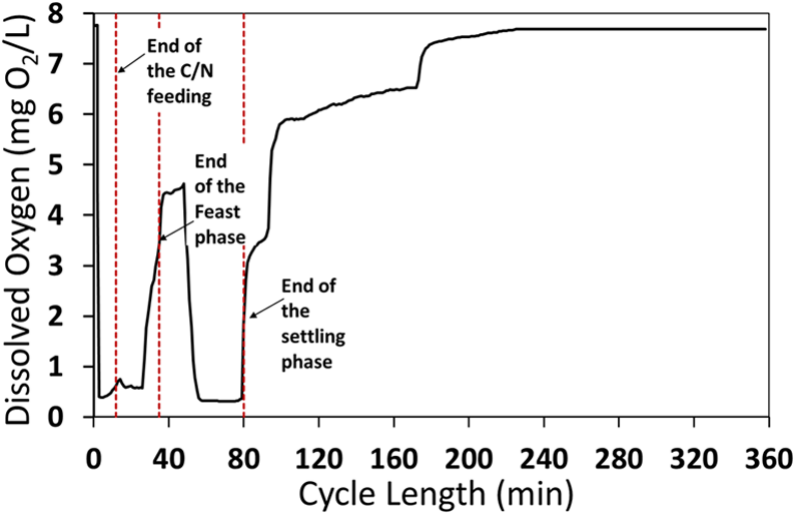
Profile of the dissolved oxygen (DO) concentration during a typical SBR cycle (6 h) fed with the RP-fermented 1 stream at an applied OLR of 2.12 gCOD_ACIDS_/Ld.

When the RP-fermented 1 stream was used as the feeding solution, the OLR applied to the SBR accounted for 2.12 gCOD_ACIDS_/Ld. The profile of the DO concentration (Figure 2) was indicative of the microbial activity rate, depending on the availability of COD in the reactor. Notably, the fraction of organic acids over _S_COD_TOT_ was around 50%, likely representing most of the readily biodegradable COD (rbCOD) (Table 2). In particular, the DO concentration suddenly decreased corresponding to the beginning of the SBR cycle (i.e., the beginning of the feeding phase), and a sharp increase in the DO concentration was observed corresponding to the end of the feast phase, indicating the depletion of the rbCOD, which was exhausted before the start of the settling phase. During this latter, the DO concentration quickly decreased due to the lack of aeration. After the settling phase, most of the soluble residual slowly biodegradable COD (sbCOD) was withdrawn with the clarified supernatant, and the remaining fraction in the reactor accounted for 194 ± 26 gCOD per cycle. At the end of the SBR cycle, the DO concentration resumed at the initial values (almost 8 mg/L).

**Figure 3:**
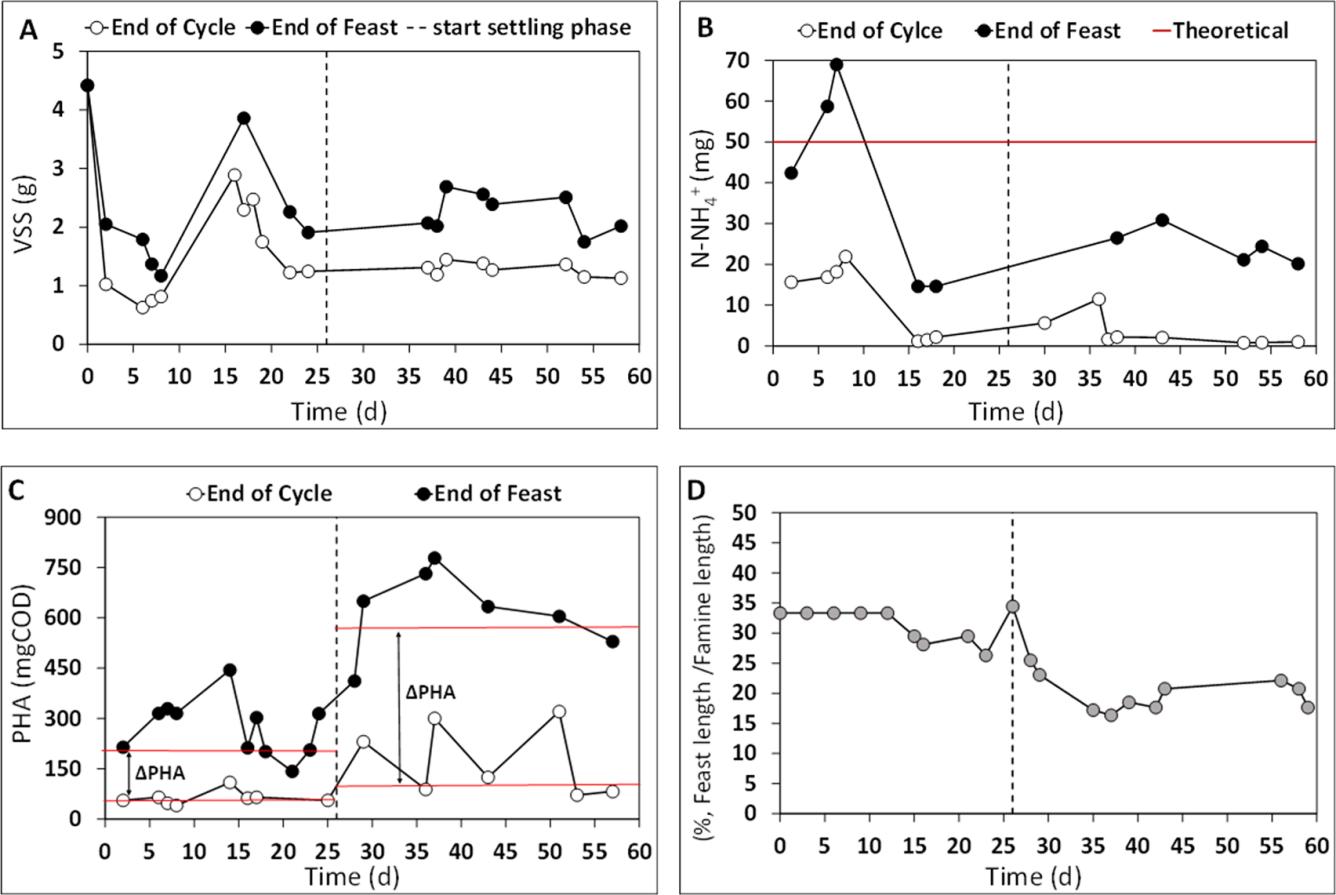
Trend of VSS profile (A), N-NH_4_^+^ profile (B), PHA profile (C), and duration of the feast phase with respect to the length of the famine phase (D), during the SBR operation fed with the RP-fermented 1 stream at an applied OLR of 2.12 gCOD_ACIDS_/Ld.

To establish a high microbial selective pressure in the SBR, a settling phase was introduced starting from day 25 of the operation resulting in an SRT equal to 1 day and an HRT equal to 0.5 days.^28^ The imposed operating parameters allowed triggering a good selective pressure under the F/F conditions, as confirmed by the profile of the quantity of VSS, N-NH_4_^+^, PHA, and the duration of the feast phase with respect to the length of the famine phase (Figure 3). The trend of VSS (Figure 3A) highlights that the value measured at the end of the famine phase (1.58 ± 0.15 gVSS) was always lower than that detected at the end of the feast phase (2.33 ± 0.14 gVSS), and this was likely because microbial growth occurred simultaneously with polymer storage (feast phase). Furthermore, during the famine phase, microbial growth from the oxidation of the intracellular polymer as a carbon source caused a substantial decrease in the stored PHA as well as an effective nitrogen consumption. Indeed, as reported in Figure 3B, the amount of ammonia detected at the end of the feast phase (23.4 ± 2.1 mg N-NH_4_^+^) was significantly higher than that measured in correspondence to the end of the cycle (2.9 ± 0.8 mg N-NH_4_^+^). Also, the value of nitrogen supplied per cycle was equal to 50 mg/cycle; therefore, the amount of nitrogen consumed by microorganisms during the feast phase accounted for 26.6 ± 3.6 mg of N-NH_4_^+^, thus clearly indicating the occurrence of microbial growth also during the feast phase. The presence of nitrogen (2.5 ± 0.1 gN-NH_4_^+^/L, Table 2) in the RP-fermented 1 mixture could negatively affect the SBR performance for the microbial selection of PHA-storing microorganisms.^39^ Here, the ratio between the C and N source, referred to as the soluble COD expressed as the fraction of the carboxylic acids, accounted for approximately 14 (gCOD/gN). However, a high selective pressure was established in the reactor (Figure 3C), as demonstrated by the considerable difference between the amount of the polymer measured at the end of the feast phase and at the end of the cycle (ΔPHA). During the operating period from day 0 until day 25, characterized by the absence of a settling phase, an average (ΔPHA) value of 205 ± 19 mg COD was achieved, and it increased up to 525 ± 27 mg COD with the addition of the settling phase in the SBR cycle. These findings suggest that the operating conditions imposed on the reactor had a positive effect on the enrichment of PHA-storing bacteria and on polymer accumulation. These results were also confirmed by the duration of the feast phase with respect to the length of the famine phase (Figure 3D). The feast/famine length was on average equal to 22 ± 1% (during the working period from day 25 onward), and this value falls within the range identified for the optimal performance of a selection process under aerobic dynamic feeding (ADF) conditions with MMC consortium (equal or less than 25%).^40^

### 3.3 Microbial Selection in the Lab-Scale SBR Fed with RP-Fermented 2 at Two Different OLRs (2.12 and 4.25 gCOD_ACIDS_/Ld)

The lab-scale SBR was fed with RP-fermented 2 at two OLR values to evaluate the effect of this parameter on the selection of the PHA-storing consortium. The two runs with the OLR equal to 2.12 gCOD_ACIDS_/Ld (run1) or 4.25 gCOD_ACIDS_/Ld (run2) lasted 42 and 57 days, respectively. Differently from RP-fermented 1, RP-fermented 2 was characterized by the absence of propionic acid and the presence of caproic acid (Table S1); therefore, the consumption of each acid was deeply analyzed during the SBR cycle. Figure S2A points out the residual amount of the overall carboxylic acids measured at different moments (i.e., end of the feeding phase, end of the feast phase, and end of the cycle) of the reactor cycle for both runs 1 and 2. In correspondence to the end of the feeding phase, the acids present in the reactor accounted for 163.4 ± 19.7 and 419.5 ± 35.2 mg COD for runs 1 and 2, respectively, resulting in a percentage of acid consumption during the feeding phase of 69.2 ± 1.5 and 60.4 ± 2.1 (%, COD_ACIDS_/_S_COD_TOT_). This confirms the high metabolic response provided by the enriched consortium and reveals the consumption of a substantial fraction of the rbCOD during the feast phase, favoring the establishment of the feast and famine conditions. Notably, the increase in the applied OLR did not negatively affect the removal of carboxylic acids during the feast phase and, in turn, the microbial selection efficiency. A similar outcome was reported for a pilot-scale SBR fed with fermented fruit waste but operated with an uncoupled carbon and nitrogen feeding strategy.^41^ As for the consumption of each acid (i.e., acetic, butyric, valeric, and caproic) during the feeding phase, the highest residual amount was represented, as expected, by caproic acid (Figure S2B), which was a medium-chain carboxylic acid, and its oxidation was kinetically disadvantaged over the oxidation of the SCA contained in RP-fermented 2.^42^ Importantly, the organic acids supplied at the beginning of the cycle were completely depleted by the end of the feast phase (Figure S2A), clearly indicating the alternance of the presence (feast) and lack (famine) of rbCOD during the SBR cycle.

**Figure 4:**
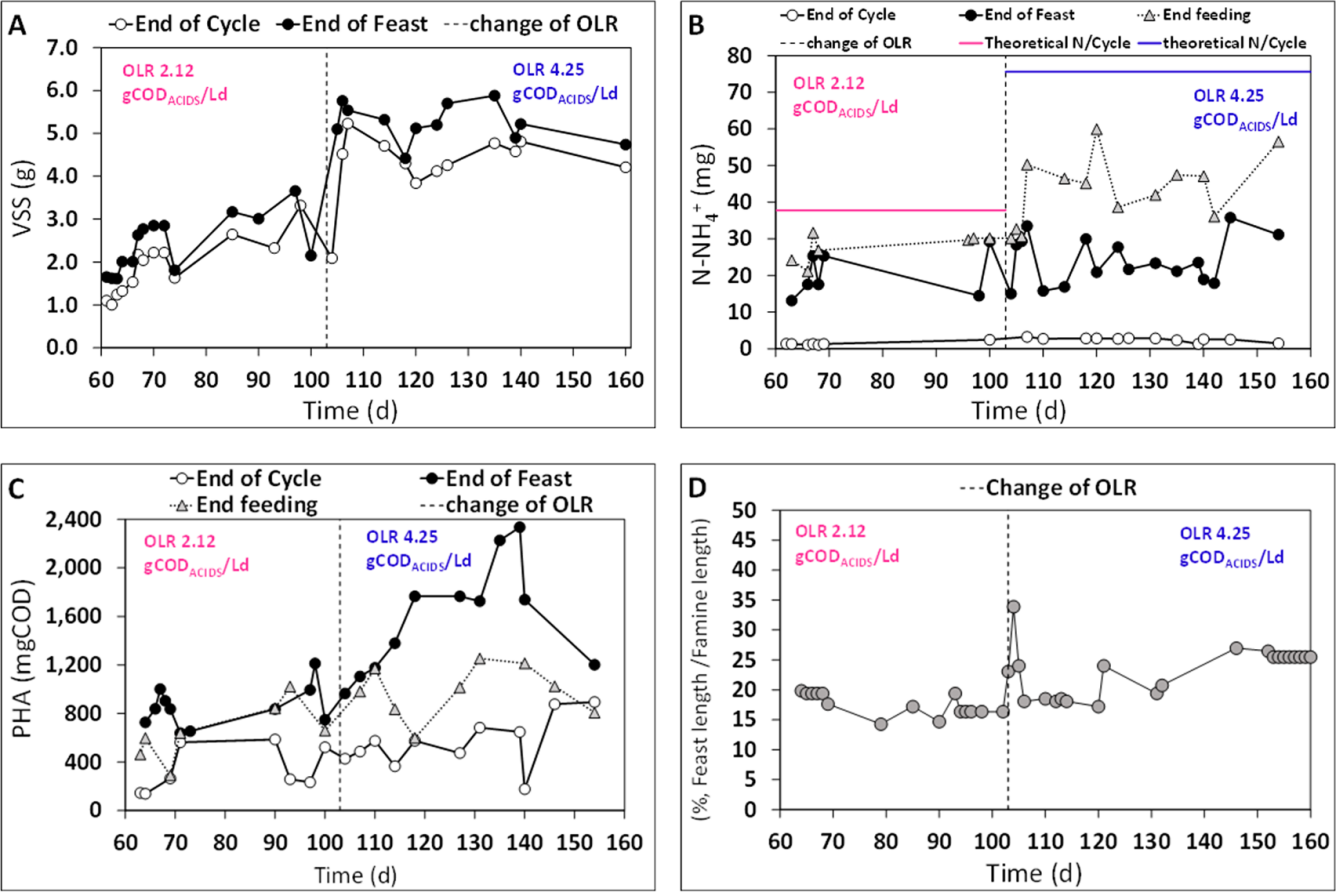
Trend of VSS profile (A), N-NH_4_^+^ profile (B), PHA profile (C), and duration of the feast phase with respect to the length of the famine phase (D), during the SBR operation fed with the RP-fermented 2 at two applied OLR values of 2.12 and 4.25 gCOD_ACIDS_/Ld.

Figure 4A shows that the VSS values detected at the end of the feast phase (2.6 ± 0.2 and 5.2 ± 0.2 gVSS, for runs 1 and 2, respectively) were higher than the values measured at the end of the famine phase (2.1 ± 0.2 and 4.4 ± 0.1 gVSS for runs 1 and 2, respectively) at both the OLR values investigated. As expected, the VSS values measured in run2 were always higher than values in run1 as a direct effect of the higher applied OLR. The trend of the N source during characteristic moments of the SBR working cycle is reported in Figure 4B. The theoretical amount of supplied nitrogen was identified by the ammonia concentration in the RP-fermented 2 and accounted for 37.8 and 75.6 mg of N per cycle for runs 1 and 2, respectively. In addition, the amount of nitrogen consumed during the feast phase accounted for 17 ± 2 and 51 ± 1 mgN for run1 and run2, respectively, and this corresponded to about 45 and 67% of the consumption of the theoretical value of ammonia supplied during the feeding phase. The ammonia measured at the end of the famine phase was similar in both runs (1.7 ± 0.2 and 2.5 ± 0.2 mgN), resulting in consumption during this specific phase of the theoretical supplied ammonia of 50 and 29% for runs 1 and 2, respectively. However, despite the occurrence of partial biomass growth during the feast phase, a high difference (ΔPHA) between the amount of PHA measured at the end of the feast phase and at the end of the famine phase was detected, corresponding to 425 ± 38 and 750 ± 41 mg COD for runs 1 and 2, respectively. Figure 4C also reports the PHA amount detected corresponding to the end of the SBR feeding phase, which was equal to 638 ± 94 and 1040 ± 89 mg COD for runs 1 and 2, respectively. In particular, for run1, this value was very similar to the amount of polymer (812 ± 44 mg COD) measured at the end of the feast phase, whereas a higher value (1571 ± 111 mg COD) was obtained in run2 in correspondence to the end of the feast phase due to the higher amount of acids provided during the feeding phase. Overall, concerning the feast phase, the calculated storage yields (0.43 ± 0.06 and 0.53 ± 0.03 COD_PHA_/sCOD_TOT_ for run1 and run2, respectively) were significantly higher than the growth yields (0.17 ± 0.03 and 0.28 ± 0.03 COD_XA_/sCOD_TOT_ for run1 and run2, respectively), pointing out that the rbCOD consumed during the feast phase was mostly used for polymer storage.

Moreover, the increase of (ΔPHA) as a direct response to the higher applied OLR confirmed that the settling strategy and the adopted process parameters were efficient in selecting the PHA-storing microorganisms under a coupled carbon and nitrogen feeding strategy. The length of the feast phase was determined from the DO profile, whose trend for both runs (Figure S3) was very similar to that obtained during the experiment conducted with RP-fermented 1 (Figure 2). For both runs, the duration of the feast phase indicates the establishment of the required selective pressure for the enrichment of PHA-storing microorganisms. Indeed, the feast length relative to the length of the famine phase (Figure 4D) obtained during run1 was lower than 20% (i.e., 17 ± 1%) and increased up to approximately 23 ± 2% when the OLR was imposed at 4.25 gCOD_ACIDS_/Ld, thereby representing a short fraction of the working cycle in both runs.

### 3.4 Comparison of the SBR Performance Operated under Different Feeding Conditions

The suitability of the RP-fermented mixtures collected from the AF-pilot reactor for the microbial selection toward PHA-storing microorganisms in the lab-scale SBR has been evaluated by taking into account key process parameters.

**Figure 5:**
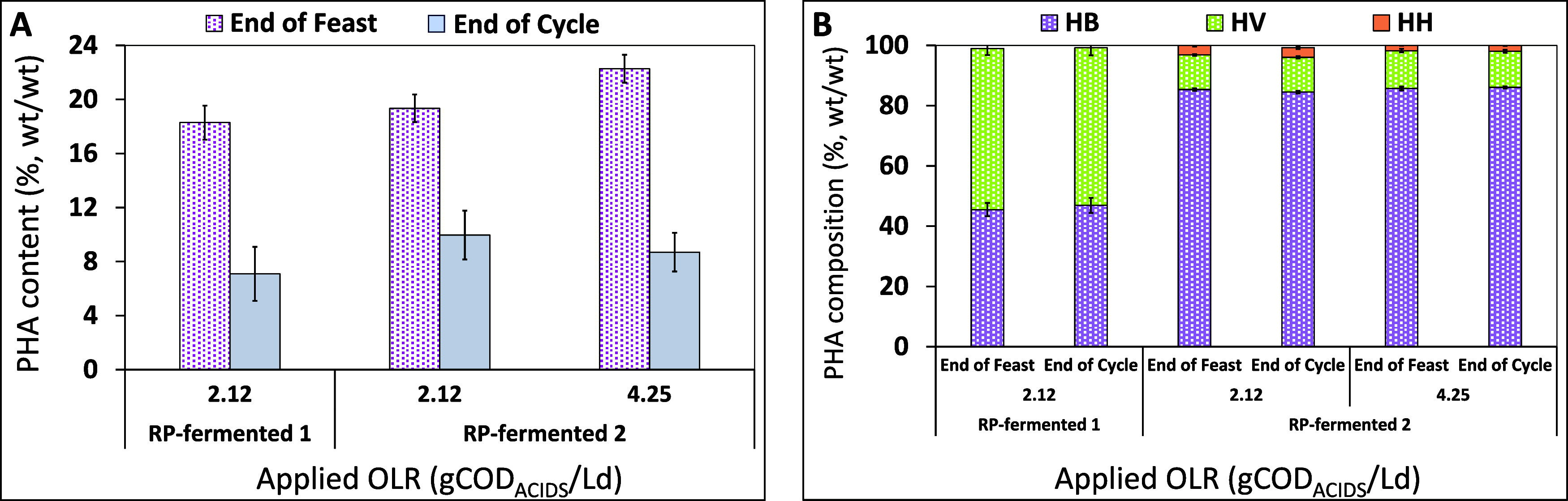
Intracellular PHA content (A) and composition of the stored polymer in correspondence to the end of the feast phase and the end of the cycle (B) obtained in the SBR fed with RP-fermented 1 and RP-fermented 2. Error bars represent ± standard error of average measurements (*n* > 10) taken throughout each SBR run.

Figure 5A reports the intracellular PHA content, which reached comparable values of 18 ± 1 and 19 ± 1 (%, wt/wt) with RP-fermented 1 and RP-fermented 2, respectively. In addition, when the OLR was increased with the RP-fermented 2, the intracellular PHA content measured at the end of the feast phase also slightly increased accounting for 22 ± 1 (%, wt/wt). Furthermore, all runs conducted with different RP-fermented mixtures highlighted the consumption of the polymer during the famine phase (Figure 5A), which is characteristic of the PHA-storing consortium operated under the F/F regime.^39^ The intracellular PHA values at the end of the famine phase were 7 ± 2 (%, wt/wt) (RP-fermented 1) and about 10 ± 3 and 9 ± 1 (%, wt/wt), with RP-fermented 2 at an applied OLR of 2.12 and 4.25 gCOD_ACIDS_/Ld; respectively. Overall, it is important to mention that the intracellular polymer content is conventionally maximized through accumulation batch tests (not investigated in this study), for which it would be relevant to evaluate the role of the high ammonia concentration contained in the RP-fermented mixtures (Table 2). Indeed, even though a recent study demonstrated MMC-PHA production at a pilot scale with salmon peptone under high ammonia concentration and constant nutrient availability,^43^ the PHA accumulation step is typically performed under nutrient-deficient conditions. As an example, Silva and colleagues^9^ reported a high value of the intracellular PHA content (71.3%, wt/wt) during the accumulation step of successfully selected microbial cultures using a fruit pulp mixture poor in ammonia. Similarly, a substantial increase in the intracellular PHA content was obtained in batch accumulation tests performed with selected MMC by using fermented olive oil mill wastewater poor in ammonia.^28^

As expected, the different RP-fermented mixtures characterized by different acid distributions (Table 2) affected the polymer composition. Indeed, the HV content at the end of the feast phase and at the end of the cycle (accounting for 54 ± 2 and 52 ± 3%, wt/wt; respectively) obtained when the SBR was fed with RP-fermented 1 was significantly higher than the HV content of both runs conducted with RP-fermented 2 (i.e., around 12%, wt/wt). When the SBR was fed with the RP-fermented 2, a terpolymer containing the 3HHx monomer was obtained with a very low 3HHx content in the polymer stored in the two runs operated at different OLR values. In general, under all of the investigated conditions, the polymer composition did not significantly change during the SBR cycle.

**Figure 6:**
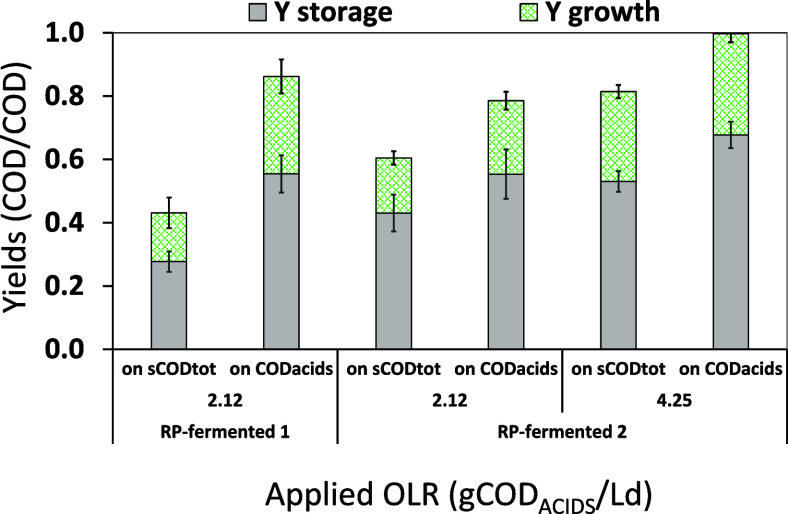
Storage and growth yields calculated in the SBR fed using the RP-fermented 1 (no pH control) and RP-fermented 2 (pH 5.90) mixtures. Error bars represent ± standard error of average measurements (*n* > 10) taken throughout each SBR run.

To assess the storage ability of the biomass selected in the SBR fed with different RP-fermented mixtures, the storage and growth yields were calculated and compared. Figure 6 points out high values of the storage yield (between 0.55 ± 0.06 and 0.68 ± 0.04 COD_PHA_/COD_ACIDS_, calculated by only taking into account organic acids for the consumed COD) for both RP-fermented mixtures. For all runs, the values of storage yield calculated with respect to the total soluble COD (between 0.28 ± 0.03 and 0.53 ± 0.03 COD_PHA_/_S_COD_TOT_) were lower than the corresponding values calculated by only taking into account the COD represented by the organic acid fraction. Notably, at the same OLR (i.e., 2.12 gCOD_ACIDS_/Ld) but with different RP-fermented streams used as feedstock, the same value of the storage yield over COD_ACIDS_ was achieved (0.55 ± 0.06 and 0.55 ± 0.07 COD_PHA_/COD_ACIDS_, for RP-fermented 1 and RP-fermented 2, respectively). In contrast, comparing the results of the storage yields on _S_COD_TOT_ (at the same OLR) it is interesting to note that the value measured during the first period with RP-fermented 1 (i.e., 0.28 ± 0.03 COD_PHA_/_S_COD_TOT_) was lower than the storage yield value detected in the second one with RP-fermented 2 (0.43 ± 0.06 COD_PHA_/_S_COD_TOT_). These findings are in accordance with the fact that RP-fermented 2 was more enriched into organic acids with respect to _S_COD_TOT_ (75% wt/wt, Table 2) than RP-fermented 1. Furthermore, with RP-fermented 2, the values of the storage yield increased as the OLR, accounting for 0.43 ± 0.05 and 0.53 ± 0.03 COD_PHA_/_S_COD_TOT_ with respect to the overall soluble COD for run1 and run2, respectively, or to 0.55 ± 0.07 and 0.68 ± 0.04 COD_PHA_/COD_ACIDS_ with respect to organic acids. This clearly underlines that the applied OLR affects the efficiency of the selective pressure, as previously reported in the literature for both real and synthetic feedstocks.^39,41^

As for the growth yields (Figure 6), at the same OLR, comparable values were determined, equal to 0.15 ± 0.05 and 0.31 ± 0.05 COD_XA_/COD (with respect to the _S_COD_TOT_ or organic acids with RP-fermented 1, respectively) and equal to 0.17 ± 0.02 and 0.23 ± 0.03 COD_XA_/COD (with respect to the _S_COD_TOT_ or to organic acids with RP-fermented 2 in run1, respectively). However, with RP-fermented 2 fed to the SBR in run2, an increase in the growth yield (up to 0.28 ± 0.03 COD_XA_/sCOD_TOT_ and 0.38 ± 0.03 COD_XA_/COD_ACIDS_) was observed. According to these data, an increase of the OLR from 2.12 to 4.25 gCOD_ACIDS_/Ld caused a metabolic shift with a higher fraction of the rbCOD removed during the feast phase being diverted toward microbial growth, likely due to the higher available ammonia concentration.

The polymer obtained at the same OLR (2.12 gCOD_ACIDS_/Ld) was extracted and characterized in terms of purity and *M_w_* (Table S2). Comparable values were obtained in terms of *M_w_* (3.39 and 3.89 × 10^5^ Da with RP-fermented 1 and RP-fermented 2, respectively), suggesting that the different compositions of carboxylic acids in the fermented mixtures did not affect the chain elongation process during PHA synthesis. Importantly, the *M_w_* and PDI values herein detected are fully comparable with data collected by previous studies, wherein the PHA-storing consortia were fed with a real feedstock such as fermented molasses (i.e., *M_w_* between 3.5 × 10^5^ and 9.0 × 10^5^ Da and PDI between 1.8 and 3.9).^17,44^

### 3.5 Synthesis of the Terpolymer in the SBR Fed with RP-Fermented 2 at Two Different OLRs (2.12 and 4.25 gCOD_ACIDS_/Ld)

**Figure 7:**
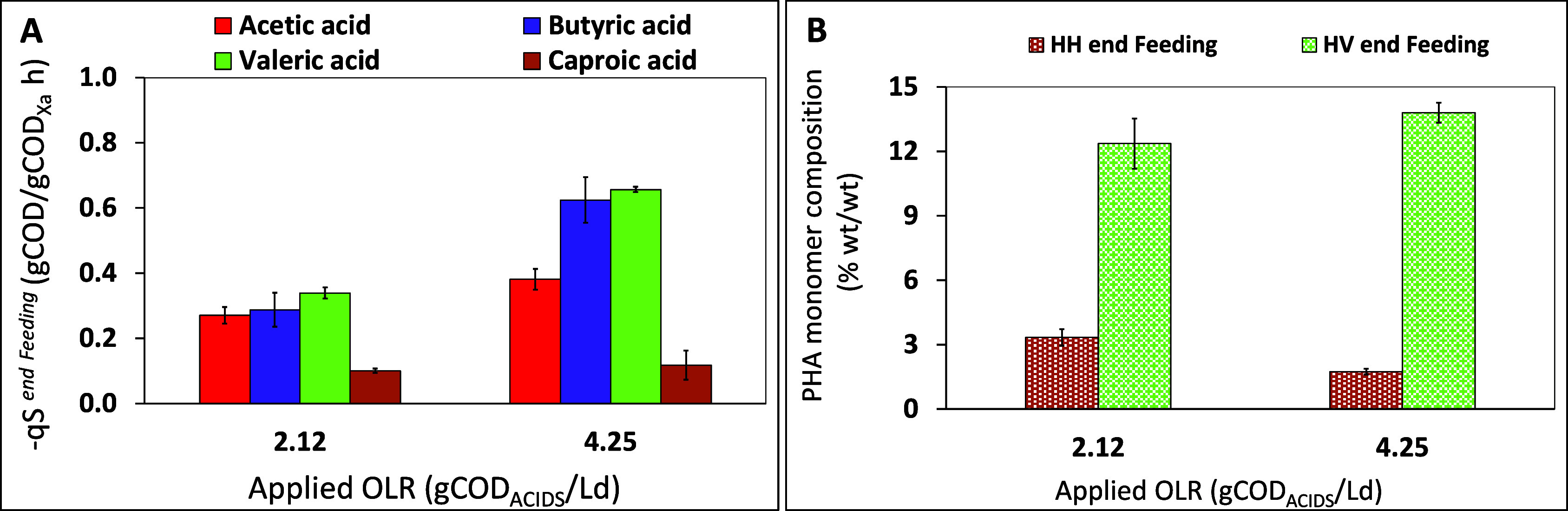
Specific acids removal rates (A) and PHA composition (B) in terms of HV and HHx content, in the SBR in correspondence to the end of the feeding phase with RP-fermented 2 at two applied OLR values of 2.12 and 4.25 gCOD_ACIDS_/Ld. Error bars represent ± standard error of average measurements (*n* > 10) taken throughout each SBR run.

A deeper analysis on the composition of the polymer obtained in the SBR fed with the RP-fermented 2 mixture was also performed to evaluate the effect of the applied OLR on the terpolymer composition. In particular, the specific substrate removal rates, measured during the feeding phase, referred to each carboxylic acid were determined for both run1 and run2, and an increase in their values was observed as the OLR increased from 2.12 to 4.25 gCOD_ACIDS_/Ld, except for the rate of caproic acid removal. Figure 7A reports the specific removal rate for acetic acid (0.27 ± 0.03 and 0.38 ± 0.03 gCOD_ACETIC ACID_/gCOD_XA_ h, for run1 and run2, respectively) and butyric acid (0.29 ± 0.05 and 0.62 ± 0.07 gCOD_BUTYRIC ACID_/gCOD_XA_ h, for run1 and run2, respectively), which significantly rose with the OLR applied to the SBR. The removal rates referred to valeric acid also presented the same trend, and the related values accounted for 0.34 ± 0.02 and 0.66 ± 0.01 gCOD_VALERIC ACID_/gCOD_XA_ h, for run1 and run2, respectively. On the contrary, the caproic acid removal rates were comparable for both runs (0.10 ± 0.01 and 0.12 ± 0.05 gCOD_CAPROIC ACID_/gCOD_XA_ h, respectively). This finding could be supported by the consideration that, when the OLR increased, the selected microorganisms preferentially consumed short-chain length acids (e.g., acetic and butyric acid) rather than caproic acid (i.e., medium-chain length acids).^42^ Moreover, the fact that the removal rate of the latter was lower than that calculated for the other acids at both investigated OLRs is likely due to the lack of caproic acid in the RP-fermented 1 stream initially used as the SBR feedstock. A slight enhancement of the HV content at the end of the feeding phase was also observed when the OLR increased (12 ± 2 and 14 ± 1% wt/wt, for runs 1 and 2, respectively), but not enough to justify the increased rate of valeric acid consumption. Indeed, the mechanisms underlying the terpolymer storage are still unclear, and this aspect needs further investigation. Differently, at higher OLR, the synthesis of the 3HHx monomer decreased (Figure 7B). In particular, the HHx monomer content was 3.3 ± 0.4 and 1.7 ± 0.1 (%, wt/wt) at the end of the feeding phase for runs 1 and 2, respectively, and these values are comparable with those obtained at the end of the feast phase and at the end of the cycle for each run (Figure 5).

### 3.6 PHA-Storing Microbiome Dynamics

**Figure 8:**
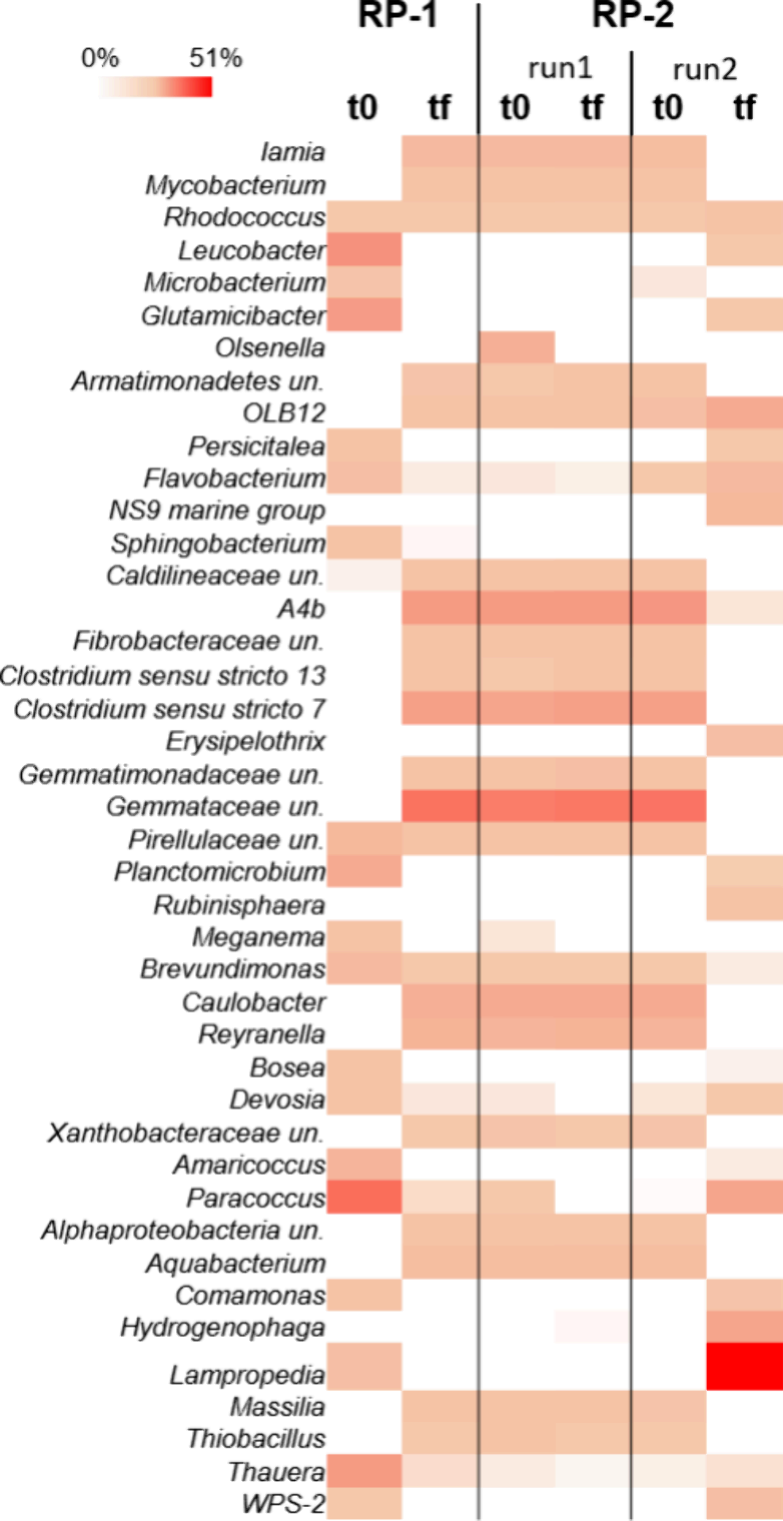
Heat map representing the relative abundance of microbial genera (≥1% relative abundance of total reads in at least one sample) in the SBR fed with different feeding steams and OLRs. The color scale ranges from 0 (white) to 51% (red) relative abundance.

The high-throughput sequencing of the 16S rRNA gene of the biomass samples collected during the SBR operation allowed us to describe the temporal dynamics of microbial community composition in response to the different applied operating conditions (Figure 8). At the beginning of the RP-fermented 1 experiment, the biomass was mainly colonized by genera *Paracoccus* (19.7% of total reads) and *Thauera* (10.1%), followed at a minor extent by *Leucobacter* (11.8%) and *Glutamicibacter* (9.7%). Although this biomass presented well-known PHA-accumulating bacteria,^45^ the operating conditions imposed to the system (e.g., feedstock rich into ammonia; settling phase and subsequent removal of the sbCOD) forced the selection of a microbiome mainly represented by members of phyla *Chloroflexi*, *Firmicutes*, *Planctomycetes*, and *Proteobacteria* constituting a putative PHA-storing bacteria abundance around 30% of total reads. In particular, the occurrence of genera *Caulobacter*, *Aquabacterium*, and *A4b* within the *Anaerolinae* class concurred with the observed PHA production (Figure 8).

The use of the RP-fermented 2 mixture allowed for the selection of biomass in run1 very similar to that observed in the run performed with RP-fermented 1 at the same OLR of 2.12 gCOD_ACIDS_/Ld. In contrast, the increase in the level of the OLR (up to 4.25 gCOD_ACIDS_/Ld) strongly affected the microbiome selection in run2. Indeed, at the end of this run with RP-fermented 2, reads affiliated with genera *Lampropedia* (up to 51% of total reads) and *Hydrogenophaga* (7.9%) were identified, which represent the PHA-storing core microbiome,^46^ in line with the highest PHA production observed in this run (Figure S4).

It is worth noting that the similar microbial community composition herein observed in the SBR fed with RP-fermented 1 and RP-fermented 2 (run1) at 2.12 gCOD_ACIDS_/Ld reflected different polymer compositions due to the ability of the same bacteria to metabolize different carboxylic acids.

For the first time, an absolute quantification of the gene involved in the PHA synthesis (*phaC* gene) in these systems was performed herein. The quantification by ddPCR revealed the highest abundance of *phaC* and 16S rRNA genes in the biomass selected during run2 with RP-fermented 2 (i.e., 1.7 × 10^8^ and 1.9 × 10^11^ gene copies/gVSS, respectively; Figure S4) in line with the highest storage and growth yields and PHA production observed. In the other analyzed samples, the *phaC* gene showed an abundance range between 2.1 × 10^4^ and 1.2 × 10^7^ gene copies/gVSS. In terms of *phaC*/16 rRNA gene ratio, the highest value was revealed in the sample collected at the end of the SBR operation with RP-fermented 1. In this sample, the estimated abundance of phaC and 16S rRNA gene was 1.1 × 10^6^ and 7.2 × 10^7^ gene copies/gVSS, respectively, revealing the highest proportion between *phaC* gene and total 16S rRNA gene, thus suggesting a high potentiality of the microbiome in PHA storage in line with the observed storage yields. However, although the *phaC*/16 rRNA gene ratio was low at the end of run2 with RP-fermented 2, in terms of absolute quantification, the presence of the *phaC* gene was the greatest (by two orders of magnitude) allowing for the highest observed PHA production.

## 4 Conclusions

RP byproduct was fermented in a pilot-scale bioreactor under either no pH control conditions (RP-fermented 1 mixture) or with the pH controlled at 5.90 (RP-fermented 2 mixture). A high selective pressure toward PHA-storing microorganisms was established in a lab-scale SBR fed with both fermented mixtures. An OLR of 2.12 gCOD_ACIDS_/Ld was applied when using the RP-fermented 1 mixture, containing SCAs with both even and odd number of carbon atoms, and a P(3HB-co-3HV) copolymer was obtained. The HV content accounted for 54 ± 2 (%, w/w), with a molecular weight of 3.39 × 10^5^ Da and a PDI value of 2. Caproic acid was contained in the RP-fermented 2 mixture, and a terpolymer including the 3HHx monomer was synthesized. The latter presents elastomeric properties and can be used in a larger set of applications than the PHB homopolymer and the P(3HB-co-3HV) copolymer. For this reason, the terpolymer (still poorly investigated with MMC) has attracted considerable attention in the plastic industry, especially in the food packaging sector. When the SBR was fed with the RP-fermented 2, two OLR values of 2.12 gCOD_ACIDS_/Ld (run1) and 4.25 gCOD_ACIDS_/Ld (run2) were investigated, and a storage yield of up to 0.68 ± 0.04 COD_PHA_/COD_ACIDS_ was obtained in run2. A very similar microbiome composition (mainly colonized by members of phyla *Chloroflexi*, *Firmicutes*, *Planctomycetes*, and *Proteobacteria*) was detected by changing the SBR feedstock from RP-fermented 1 to RP-fermented 2 when the same OLR of 2.12 gCOD_ACIDS_/Ld was applied, highlighting the ability of the same bacteria to synthesize PHA with different structures. Overall, the obtained results indicate the suitability of the RP byproduct to be used as a promising feedstock for MMC-PHA production.

## Supporting Information Available

Additional experimental results, characterization of RP-fermented mixtures (RP-fermented 1 and 2) in terms of carboxylic acids and their consumption over time, purity and average molecular weight (*M_w_*) of the extracted PHA, abundance of phaC and 16S rRNA genes in the selected microbial cultures under the feast and famine regime (PDF)
