# Supplementary material for: Pilot-Scale Acidogenic Fermentation of Reground Pasta Byproduct for Polyhydroxyalkanoate Production with Mixed Microbial Cultures
Source: ACS Sustain Chem Eng. 2025 Feb 20;13(8):3024–35. doi: 10.1021/acssuschemeng.4c03754 (PMC11881138; doi:10.1021/acssuschemeng.4c03754)
Supplement: Supplementary file 1 — sc4c03754_si_001.pdf [file sc4c03754_si_001.pdf]

Supporting Information

Pilot-Scale Acidogenic Fermentation of Reground Pasta Byproduct for Polyhydroxyalkanoate Production with Mixed Microbial Cultures

Gaia Salvatori[a], Angela Marchetti[a], Anna Maria Russo[a]; Jesus Rodriguez[b,§], Vadim Scerbacov[b], Francesco Fianelli[b], Sara Alfano[a], Simona Crognale[c,d], Alessio Massimi[c], Simona Rossetti[c], Giacomo Canali[e], Tiziana De Micheli[e], David Bolzonella[f]; Marianna Villano[a,g,*]

[a]Department of Chemistry, Sapienza University of Rome, P.le Aldo Moro 5, 00185 Rome, Italy

[b]InnovEn Srl, Via San Giovanni Lupatoto 119, 37134 Verona, Italy

[c]Water Research Institute (IRSA), National Research Council (CNR), Via Salaria km 29300, 00015 Monterotondo (RM), Italy

[d]National Biodiversity Future Center (NBFC), Palermo, Italy

[e]Barilla G. e R. Fratelli – Società per Azioni – Socio Unico, Via Mantova 166, 43122 Parma (PR), Italy

[f]Department of Biotechnology, University of Verona, Via Strada Le Grazie 15, 37134 Verona, Italy

[g]Research Center for Applied Sciences to the Safeguard of Environment and Cultural Heritage (CIABC), Sapienza University of Rome, P.le Aldo Moro 5, 00185 Rome, Italy

[§]Present address: ULUU, Indian Ocean Marine Research Centre, 86 West Coast Drive, Watermans Bay, Western Australia

*Corresponding author: marianna.villano@uniroma1.it

Table of Contents

Table S1 Organic acids composition of the RP-fermented mixtures obtained during the operation of the pilot-scale fermentor.

Table S2 Characterization of PHA obtained in the SBR fed with RP-fermented 1 and RP-fermented 2 at an applied OLR of 2.12 $gCOD_{ACIDS}$/Ld.

Figure S1 Scheme of the SBR working cycle operated under the F/F regime.

Figure S2 Time course of the amount of total organic acids in correspondence to the end of the Feeding and Feast phases, and to the end of the cycle (A), and of each acid at the end of the Feeding phase (B), during the SBR operation with RP-fermented 2 fed at two applied OLR values of 2.12 and 4.25 $gCOD_{ACIDS}$/ Ld.

Figure S3 Profile of the Dissolved Oxygen (DO) concentration during a typical SBR working cycle (6 hours) with RP-fermented 2 fed at two applied OLR values of 2.12 and 4.25 $gCOD_{ACIDS}$/ Ld.

Figure S4 Abundance of *phaC* and 16S rRNA genes in the microbial culture selected in the lab-scale SBR estimated by ddPCR.

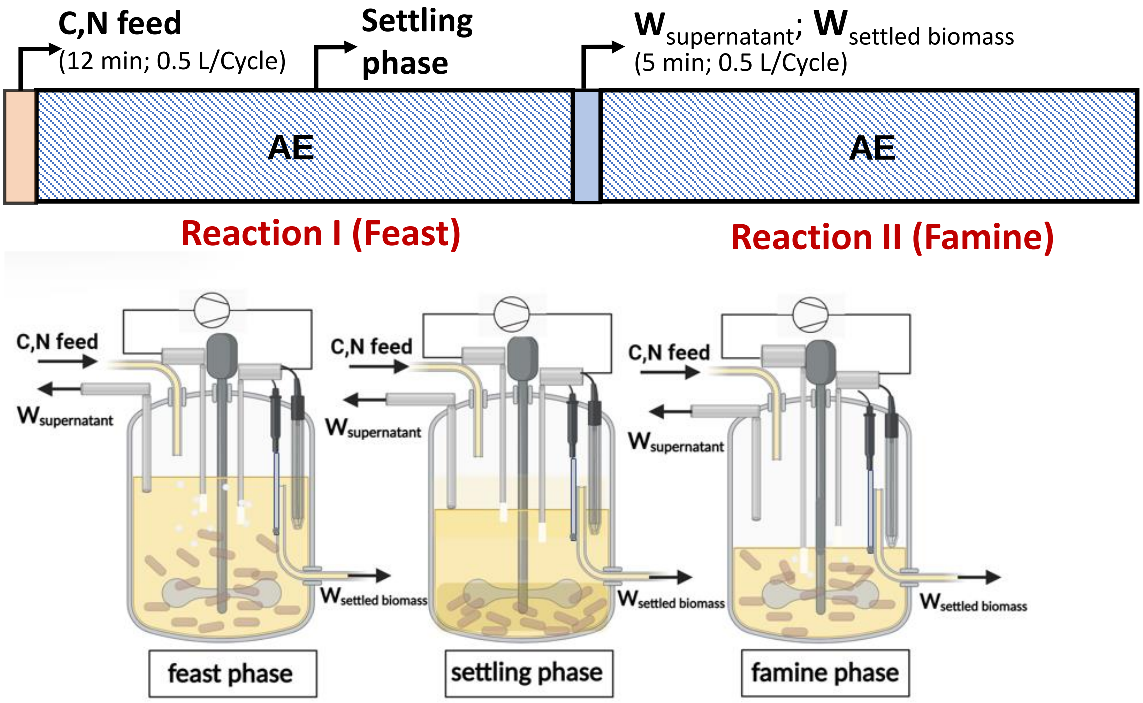

Figure S1. Scheme of the SBR working cycle operated under the F/F regime (created with Biorender).

Table S1. Organic acids composition of the RP-fermented mixtures obtained during the operation of the pilot-scale fermentor.

Fermented mixtures	Acetic acid (%, wt/wt)	Propionic acid (%, wt/wt)	Butyric acid (%, wt/wt)	Valeric acid (%, wt/wt)	Caproic acid (%, wt/wt)
**RP-fermented 1**	43.9 ± 1.7	26.4 ± 0.8	20.4 ± 1.1	9.3 ± 0.1	-
**RP-fermented 2**	21.4 ± 0.2	-	30.5 ± 0.4	25.9 ± 0.6	22.1 ± 0.8

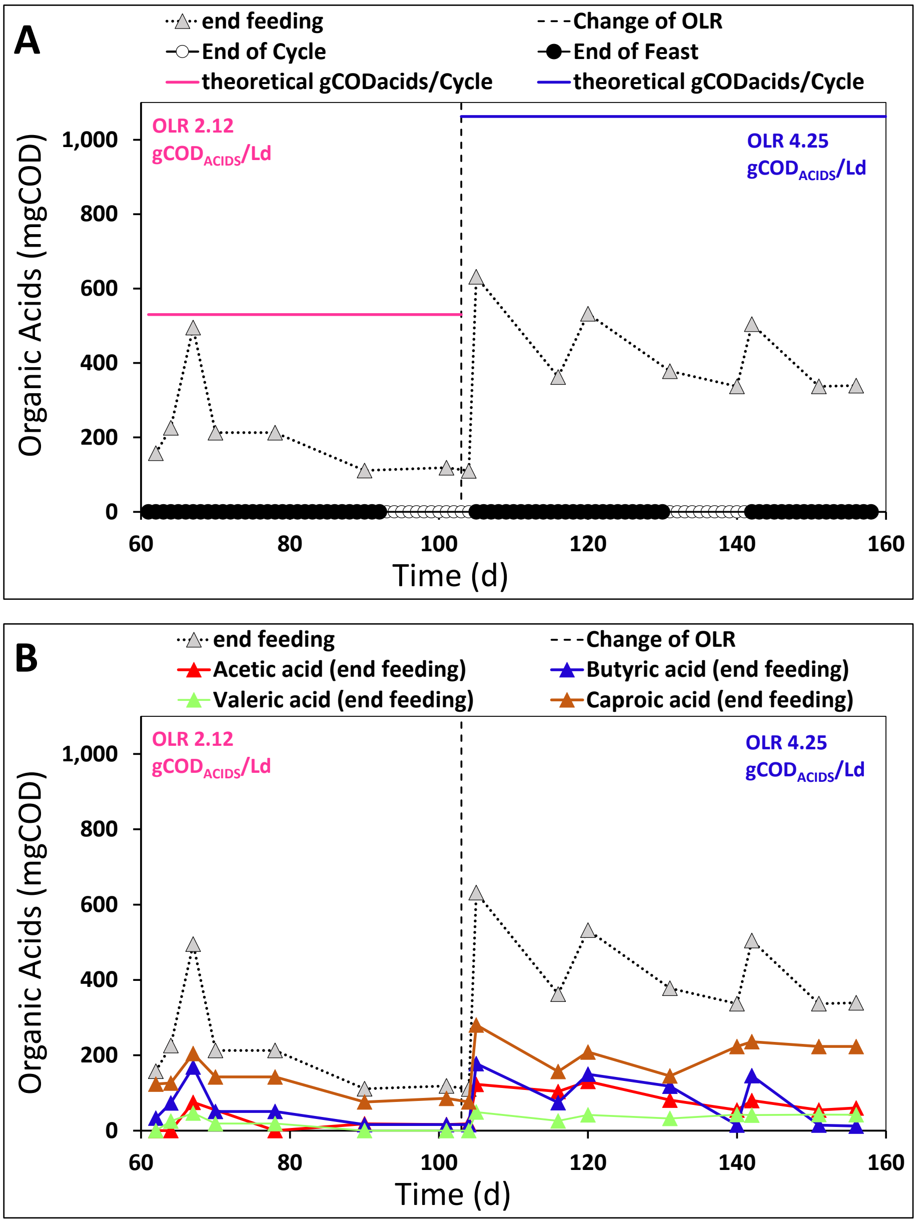

Figure S2. Time course of the amount of total organic acids in correspondence to the end of the Feeding and Feast phases, and to the end of the cycle (A), and of each acid at the end of the Feeding phase (B), during the SBR operation with RP-fermented 2 fed at two applied OLR values of 2.12 and 4.25 $gCOD_{ACIDS}$/ Ld.

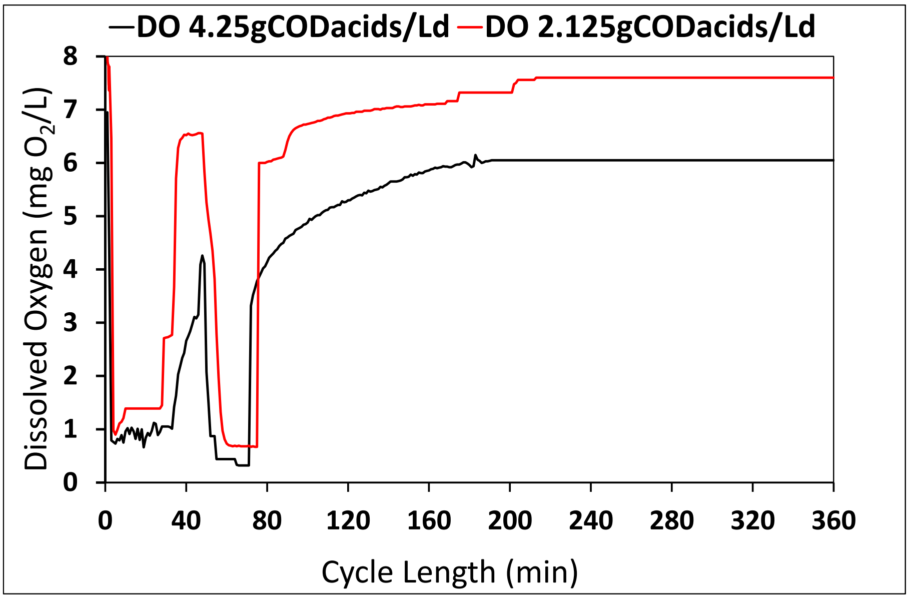

Figure S3. Profile of the Dissolved Oxygen (DO) concentration during a typical SBR working cycle (6 hours) with RP-fermented 2 fed at two applied OLR values of 2.12 and 4.25 $gCOD_{ACIDS}$/ Ld.

Table S2. Characterization of PHA obtained in the SBR fed with RP-fermented 1 and RP-fermented 2 at an applied OLR of 2.12 $gCOD_{ACIDS}$/Ld.

**Fermented mixture**	**OLR ($gCOD_{ACIDS}$/Ld)**	**Polymer composition**	**Purity (PHA %, wt/wt)**	**M_w (kDa)**	**PDI**
RP-fermented 1	2.12	P(3HB-co-3HV)	96 ± 1	339	2
RP-fermented 2	2.12	P(3HB-co-3HV-co-3HHx)	100 ± 1	389	3

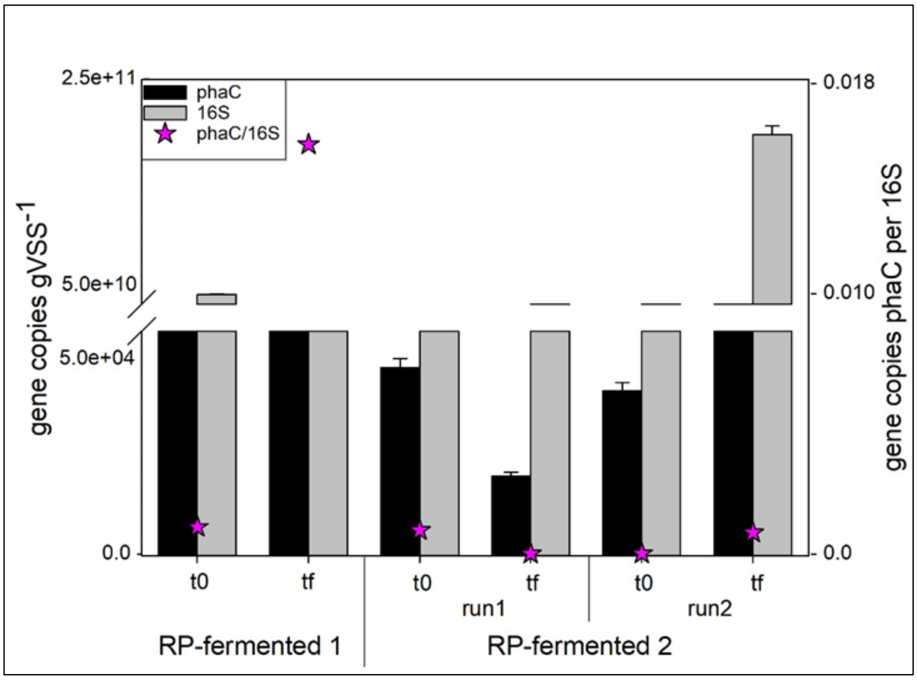

Figure S4. Abundance of *phaC* and 16S rRNA genes in the microbial culture selected in the lab-scale SBR estimated by ddPCR.
